# Factors influencing adoption of facility-assisted delivery - a qualitative study of women and other stakeholders in a Maasai community in Ngorongoro District, Tanzania

**DOI:** 10.1186/s12884-020-2728-2

**Published:** 2020-02-12

**Authors:** Paul D. Mosley, Kisiaya Saruni, Bernadetha Lenga

**Affiliations:** 1 Health Programs Coordinator, Mennonite Central Committee Tanzania, PO Box 138, Arusha, Tanzania; 2 Department of Sociology and Anthropology, Assistant Lecturer, University of Dar Es Salaam, PO Box 35043, Dar es Salaam, Tanzania; 3 Independent Consultant, PO Box 138, Arusha, Tanzania

**Keywords:** Maternal and child health, MCH, RMNCH, Antenatal care, ANC, Facility delivery, Care group, TBA, Traditional birth attendant, Tanzania, Ngorongoro, Maasai

## Abstract

**Background:**

Tanzania’s One Plan II health sector program aims to increase facility deliveries from 50 to 80% from 2015 to 2020. Success is uneven among certain Maasai pastoralist women in Northern Tanzania who robustly prefer home births to facility births even after completing 4+ ANC visits. Ebiotishu Oondomonok Ongera (EbOO) is a program in Nainokanoka ward to promote facility births through a care-group model using trained traditional birth attendants (TBAs) as facilitators. Results to date are promising but show a consistent gap between women completing ANC and those going to a facility for delivery. A qualitative study was conducted to understand psychosocial preferences, agency for decision-making, and access barriers that influence where a woman in the ward will deliver.

**Methods:**

In-depth interviews, focus group discussions and key-informant interviews were conducted with 24 pregnant and/or parous women, 24 TBAs, 3 nurse midwives at 3 health facilities, and 24 married men, living in Nainokanoka ward. Interviews and discussions were transcribed, translated, and analyzed thematically using a grounded theory approach.

**Results:**

Most women interviewed expressed preference for a home birth with a TBA and even those who expressed agency and preference for a facility birth usually had their last delivery at home attributed to unexpected labor. TBAs are engaged by husbands and play a significant influential role in deciding place of delivery. TBAs report support for facility deliveries but in practice use them as a last resort, and a significant trust gap was documented based on a bad experience at a facility where women in labor were turned away.

**Conclusions:**

EbOO project data and study results show a slow but steady change in norms around delivery preference in Nainokanoka ward. Gaps between expressed intention and practice, especially around ‘unexpected labor’ present opportunities to accelerate this process by promoting birth plans and perhaps constructing a maternity waiting house in the ward. Rebuilding trust between facility midwives, TBAs, and the community on the availability of health facility services, and increased sensitivity to women’s cultural preferences, could also close the gap between the number of women who are currently using facilities for ANC and those returning for delivery.

## Background

Two-thousand fifteen was a milestone year for the Millennium Development Goals (MDGs) to measure progress and identify successes and gaps in hitting targets on the eight development goals identified as priorities. Despite progress in many countries on MDG 5a to reduce the global maternal mortality ratio (MMR) by three quarters from 1990 to 2015, many others, most notably in Sub-Saharan Africa fell short [1, 2]. The United Republic of Tanzania’s commitment to achieving MDG Goal 5a meant reducing maternal deaths, estimated at 910 maternal deaths per 100,000 live births in 1990, to 133 maternal deaths per 100,000 live births by 2015 [3]. Based on data from the 2012 Tanzania Household and Population Census (HPC), which put MMR at 432 in 2012 [4], and the DHS estimate for 2015 of 530 [5] this ambitious target was not achieved despite remarkable progress in other reproductive, maternal, newborn, and child health (RMNCH) indicators including under-5 mortality which dropped 69% from baseline to 2013 [6]. While maternal mortality had an overall decrease of 55% since 1990, none of that change occurred in the past decade [4].

The Tanzania Ministry of Health, Community Development, Gender, Elderly and Children (MoHCDEC—formerly MOHSW) has been aware of slow progress on the maternal and newborn health indicators since 2009 when it implemented its ‘One Plan for Maternal Newborn and Child Health’ strategic plan to supplement its larger Health Sector Strategic Plan III. This plan has gone through two iterations since that time, the ‘Sharpened One Plan’, and ‘One Plan for Maternal Newborn and Child Health II’ (One Plan II) a second full iteration that extends from 2015 to 2020 with revised targets for reducing maternal mortality. Current strategic objectives for MMR have been revised to reduce maternal mortality from 410 to 292 per 100,000 live births by 2020 [7]. Operational targets related to achieving this objective are:

1. Increase antenatal care visits (4+) from 43 to 90%.
2. Increase coverage of health facility delivery from 50 to 80% of all deliveries
3. Increase coverage of deliveries attended by skilled health personnel from 51%to 80% among facility deliveries
4. Increase coverage of basic emergency obstetric care from 20% (dispensaries) to 50 and 39% (health centers) to 100%.
5. Increase coverage of comprehensive emergency obstetric care from 73 to 100% for hospitals and from 9 to 50% for upgraded health centers.
6. Increase ART coverage and retention among HIV-positive pregnant women from 79 to 100%
7. Increase postnatal care within first 48 h from 31 to 80% [7].

In northern Tanzania, particularly in rural districts of Arusha region with a high proportion of Maasai pastoralists, operationalizing these objectives has been difficult, most notably the objective of increasing the adoption of facility delivery—a practice which challenges traditional norms around childbirth in these communities. Several studies in Ngorongoro district of Northern Tanzania document the robust preference for home delivery even among women who attend 4+ ANC visits [8, 9]. The seeming paradox of high ANC and low facility birth is not unique to pastoralist groups in Tanzania, and is confirmed by studies of similar populations in Zambia [10], Kenya [11], Uganda [12] and Malawi [13].

Barriers to accessing skilled delivery services in rural Sub-Saharan Africa are well documented and commonly cited factors include: fear of mistreatment or abuse in facilities [14, 15], long distance and difficulty in access [9], poverty and education [9], lack of decision-making power and support [8, 16], other cultural issues such as birth position and preference for traditional birth attendants [13, 17], lack of knowledge of risk and promotion of health seeking behavior [18].

Scaling-up primary care services to increase access in rural areas has been a focus of the health sector since 2000 [1]. Solutions to overcome gaps in quality at points of service such as piloting respectful maternity care programs [14] and other rights-based approaches to maternal health [19, 20] have also been tested at the national level. But addressing the challenge of entrenched preferences for accessing traditional care modalities requires behavior change interventions that recognize not only the medical, but also psychosocial needs of women during pregnancy and childbirth. Bradley et al. [14] documents well the gap in provision of such services by midwives in health facilities who must manage the tension between ‘medical and social models of birth.’ “The false compartmentalization of technical quality and safety from the interpersonal aspects of care has done women in resource-poor settings a considerable disservice” [14]. According to Bradley, this ‘false compartmentalization’ accounts for the seemingly paradoxical phenomenon of pregnant women accessing ANC services but declining facility deliveries.

Naiboisho Development Initiative (NDI) is a local civil society organization that has been implementing the Ebiotishu Oondomonok Ongera (EbOO) project since November 2017. The project seeks to reduce maternal and child mortality in Nainokanoka ward using the Government of Tanzania’s One Plan II operational objectives to increase the number of women completing antenatal care (ANC) visits and going to a facility for delivery. To promote adoption of these practices, and particularly to address women’s preferences in that community for traditional home births, EbOO uses a ‘care group’ approach facilitated by trained traditional birth attendants (TBAs).

The use of the Care Group model to disseminate best practices in maternal health is based on its success in similar contexts in 28 countries [21]. A care group has been defined through best practices as:

“…a group of 10–15 volunteer, community-based health educators who regularly meet together with project staff for training and supervision. Care Groups create a multiplying effect to equitably reach every beneficiary household with interpersonal behavior change communication. They also provide the structure for a community health information system that reports on new pregnancies, births and deaths detected during home visits [22].”

The use of TBAs as care group volunteers is a context-specific innovation to leverage the trust that these women have in the community to promote antenatal care, including accompanying women to ANC visits and escorting them to facilities at the time of delivery. In conformity with current Government of Tanzania protocol, TBAs are supported to play a role as advocates for safe motherhood practices—to encourage women to complete ANC visits and have a facility delivery [23]. They are not allowed to assist in facility deliveries, although in Nainokanoka ward, some attending midwives at the three health facilities do allow TBAs to be in the delivery room and provide emotional support to the mother.

The EbOO project has 6 care groups serving the three sub-villages in the ward with 75 trained TBA volunteers who visit an average of 225 pregnant women in the ward each month through home visits. They are trained to escort women to ANC visits, provide education on breast-feeding, identify pregnancy warning signs, advocate for facility deliveries, help prepare a birth plan, and accompany those who choose to have facility deliveries when labor begins.

**Fig. 1:**
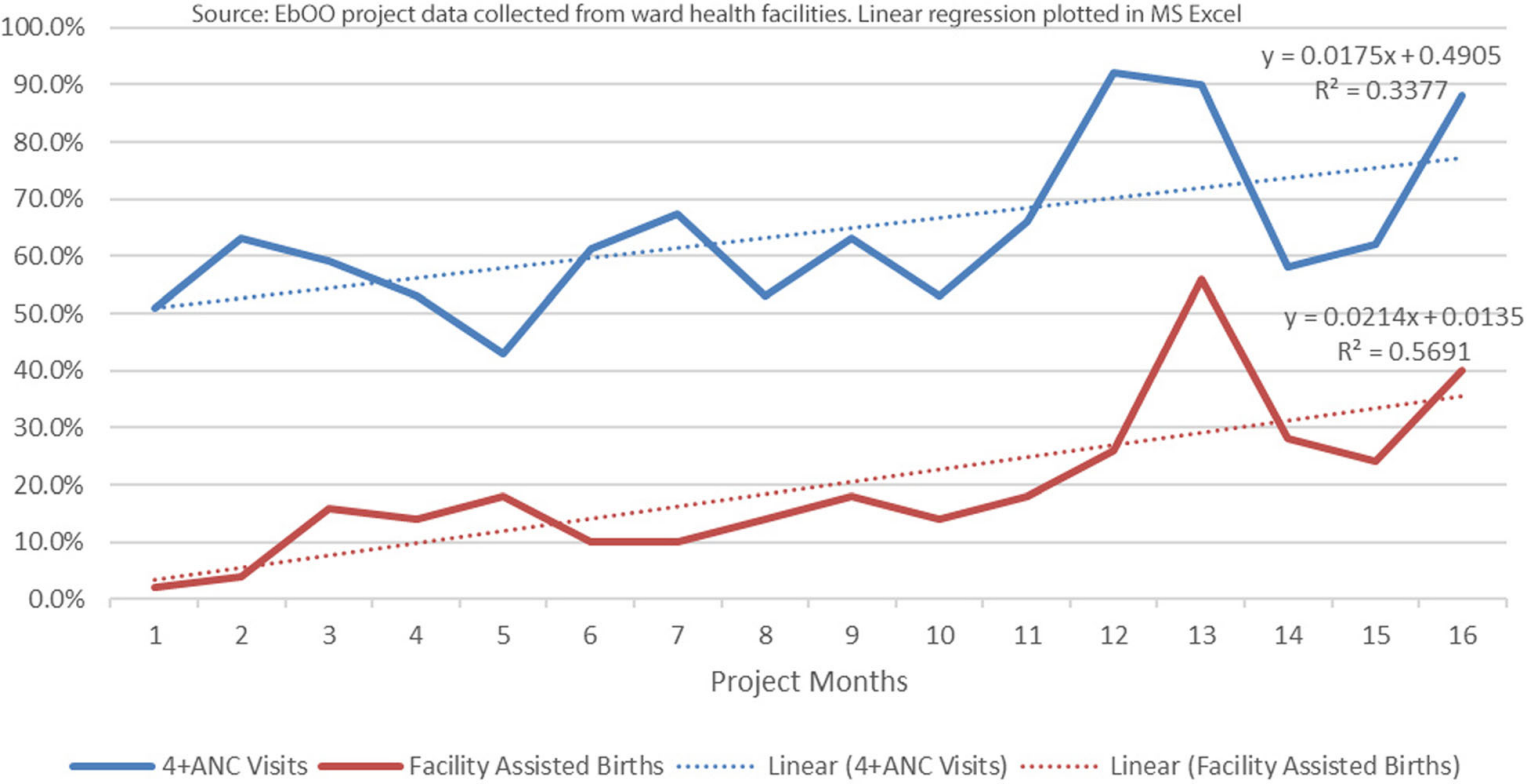
Percent Change in 4 + ANC Visits and Facility Assisted Births in Nainokanoka Ward since EbOO Project Initiation

Consistent with findings of several studies in Ngorongoro district [8, 9, 19], where Nainokanoka ward is located, project baseline data indicated a much higher rate of women attending 4+ ANC visits (51%) than facility deliveries (2%) at baseline. In the following 15 months since project implementation, both indicators increased (Fig. 1) but the gap between them remains proportionally constant. This finding contrasts significantly with the Tanzanian national context where the 2015 DHS reported attendance at 4 + ANC to be 50.7% — 12 points below facility births reported at 62.6% [5]. Anecdotally, care group volunteers confirmed that women in the ward had strong psychosocial preferences for a traditional home delivery and the gap was not fully explainable by access barriers to facility delivery.

### Objectives

In light of these findings, a qualitative study was proposed to better understand and document the reasons for the rate gap between Maasai women who complete antenatal care and those who return to a facility for delivery, in the project catchment area. Of particular interest, were psychosocial factors that explain the strong preference for a home delivery. (In this context, psychosocial factors of interest would include practices or rituals that support a woman emotionally during delivery, as well as social and power relations around those delivery practices and decision-making about place of birth.)

In addition, the study sought to document the factors that are influencing recent adopters of facility delivery to change their practice. Four research questions were proposed to guide the development of survey instruments and interview strategies for data collection.

1. Understand and document who are decision-makers for place of delivery.
2. Understand and document what (non-medical) practices are considered an essential part of a traditional home delivery compared to services provided in a clinical setting.
3. Understand and document self-reported barriers and preferences regarding facility vs. home delivery.
4. Understand and document the factors that have led to women (or key family decision makers) who have had facility deliveries, or intend to have one, to change their delivery preference.

## Methods

### Study design

This was a qualitative study conducted in Nainokanoka ward in Ngorongoro district, Tanzania, the site of NDI’s EbOO project. Data collection was in the form of in-depth interviews (IDIs), focus group discussions (FGDs) and key-informant interviews, with individuals and groups identified as relevant stakeholders in decisions about where a woman will deliver in the ward. This included pregnant and/or parous women, TBAs, husbands and male elders, and ward health facility nurse midwives. The study employed a ‘grounded theory’ approach to analyze interview and focus group data that was collected. Grounded theory is an inductive analytical framework which begins with data collection. Although research questions identified in the study objectives provide a deductive framework that shape the creation of survey instruments, it is inductive analysis of IDIs and FGDs that identify emerging patterns in the data which are developed into a theory. The emergent theory can then suggest directions for further inquiry to strengthen and confirm it.

### Study site

Nainokanoka ward is one of 11 wards in the Ngorongoro Conservation Area (NCA). A ward is a third-tier administrative zone in Tanzania, with a population between 8000 and 15,000. Wards are subsumed into districts, which in turn are subsumed into regions. Nainokanoka ward is 1109 km^2^ and populated by Maasai pastoralists who are permitted to live in the NCA with certain restrictions on farming and land use [24]. The 2017 District Population and Livestock Census counted 14,166 people living in the ward (7419 females, 6747 males), approximately 50% of whom are under the age of 14 [24]. Under-five mortality in rural Ngorongoro district where the ward is located counted 39 deaths per 1000 live births [7], and MMR was estimated at 585 maternal deaths per 100,000 live births in the 2012 HPC [4]. Based on the crude birth rate for the region (43 live births per 1000 population [25]), there are an estimated 50 births per month in the ward.

**Fig. 2:**
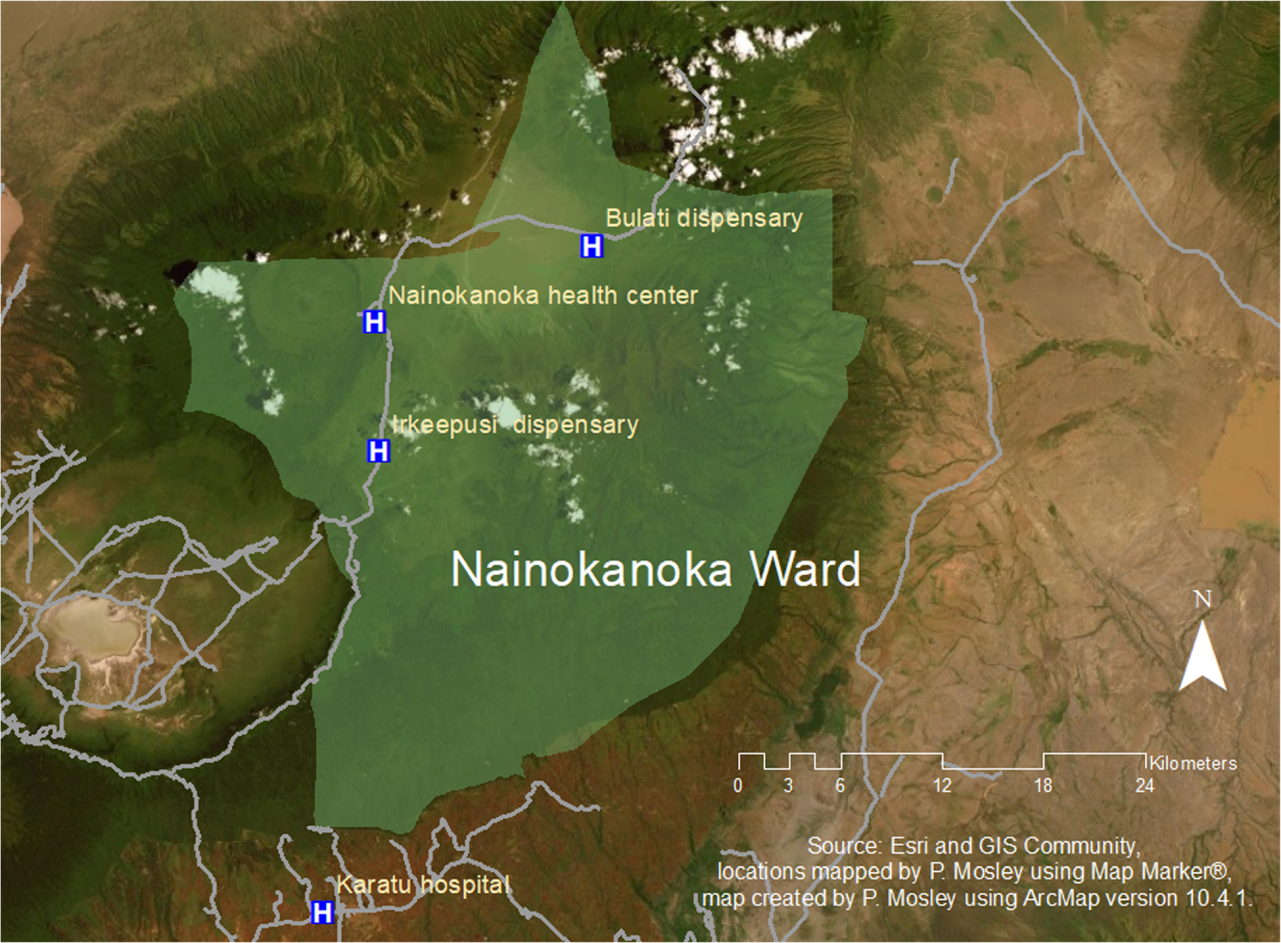
Nainokanoka Ward Health Facilities

Nainokanoka ward is served by two dispensaries and one second tier health center located in 3 sub-villages: Irkeepusi, Nainokanoka, and Bulati (Fig. 2). The three sub-villages are approximately 15 km apart along the only road in the ward. All three facilities offer ANC and skilled delivery services with basic emergency obstetric care. Nainokanoka sub-village, in the center of the ward, is the location of the 2nd tier health center which does offer referral service (with ambulance) to a tertiary care center 80 km away to the South, in the town of Karatu — the nearest location for comprehensive emergency obstetric care. According to the ward medical officer, there are 4 clinical officers (at least 1 at each facility), 5 nurses, 2 medical attendants, 1 pharmacist and 1 lab technician who work in the 3 health facilities in the ward. There is no maternity waiting house at any of the health facilities.

### Study sample

The decision criteria for sample size in a qualitative study is not standardized. Generally the strongest empirical justification for a qualitative sample is achieving a ‘saturation’ or redundancy of themes [26]. The assumption underlying saturation in this study is that all themes relevant to decisions and preferences around place of delivery will be presented through the 24 IDIs, 5 FGDs and 3 key-informant interviews, by 75 selected participants.

### Data collection methods

Data collection, in the form of 24 IDIs, 5 FGDs and 3 key-informant interviews involving 75 community members were completed between December 10, 2018 and January 31, 2019.

#### Study population

The study population was divided into four sub-groups identified as relevant stakeholders in delivery decisions in the ward:

1. Twenty-four pregnant and/or parous women 18–49 years old,^1^ who had had at least one birth in the past 5 years, living in the ward and currently participating in care-groups were identified by NDI care group promoters to participate in IDIs to better understand their agency and preferences about place of delivery. (One participant was found to not meet eligibility criteria based on time of last birth and was de-selected at the time of her interview.)
2. Twenty-four TBAs currently participating in the care groups and living in the ward were selected to provide insight into birth preferences and practices they observe and promote in women they assist in delivery. Candidates were identified by NDI care group promoters to participate in 2 FGDs.
3. Twenty-four married men including male community leaders living in the ward who were fathers of at least one child were selected to understand their perception of norms around decision-making for delivery, financing, distance, as well as their own birth preferences for their wives. Candidates were identified by NDI staff to participate in 3 FGDs.
4. Three nurse midwives were selected, one from each of the three ward health facilities, for key-informant interviews to follow-up on issues raised by participants in the IDIs and FGDs.

#### Sampling approaches

A purposive sampling technique was employed for all populations to best represent an equal cross-section of three sub-villages in the ward. Eight participants in each sub-population were chosen from each of the three sub-villages in the ward (Irkeepusi, Bulati, Nainokanoka). For women participating in IDIs there were also criteria for representation based on age, parity, and household distance from a delivery facility. Several multiparous women who had had both facility and traditional delivery were also recruited to provide insight into their experience with each.

Purposive sampling was also used in FGDs to include TBAs from all sub-villages as well as several who had experience attending both facility and home deliveries. Married men with at least one child who were selected to participate in FGDs were selected proportionally to represent each sub-village.

#### Data collection tools

IDI and FGD questions were developed in collaboration with the project team who are themselves Maasai, living in the community, and familiar with the context. IDIs and FGDs with women were facilitated by an experienced female Maasai researcher (BL) who conducted them in the Maa language. Men’s FGDs were facilitated by a male Maasai researcher (KS) in the Maa language as well. All IDIs and focus groups were recorded then transcribed into Maa and translated into Kiswahili and English for coding and analysis. Translations were reviewed by two Maa speaking researchers for accuracy. Key-informant interview questions were developed by study authors based on initial analysis of data from IDIs and FGDs. Key-informant interviews were conducted in Swahili by a Swahili speaking researcher (BL), and transcribed and translated into English.

### Ethical considerations

*Please refer to DECLARATIONS for details on Ethical Approval and Consent to Participate.*

### Analytical approach

Transcribed and translated data from de-identified key informant discussions, FGDs and IDIs, were analyzed and coded independently by two researchers—one, a Maasai from the community (KS), and one American (PM). Initial coding was done manually to identify major themes. Once major themes and subthemes were identified and compared, a common codebook of themes was created based on consensus of the researchers, and agreement from key EbOO project informants. The data was then recoded using NVIVO 12 analytical software for further analysis in preparation for use in publication. From the coded data, a theory was constructed, based on synthesis and deductive analysis by the two principle investigators.

Results of the study were presented to key informants in the EbOO project as well as health professionals in the ward and professional colleagues involved in the project for feedback, as part of the project action-learning cycle. Feedback was used to further refine the emergent theory before final preparation of results for distribution and publication.

## Results

In individual IDIs, women were asked demographic questions about age, marital status, parity, and whether they were currently pregnant, followed by a range of questions about health seeking behavior including number of ANC visits they had attended, and whether they practiced family planning. Pregnant women were also asked about preparations they were making for their current delivery, where they preferred to have their delivery (and why) and which decision makers-were involved in that decision. In addition, all parous women (*n* = 20) were asked where they had their last delivery, who attended and what their role was, what preparations were made, who made decisions for place of delivery, what position did they give birth in, and whether they attended a postpartum clinical visit after delivery. Some descriptive statistics for women surveyed are in Table 1.

**Table 1:** Descriptive Statistics of IDI participants (Source: IDIs)

# of women interviewed	23
Age range	18–47
# of women interviewed who were married	21
# of parous women interviewed	20
# of currently pregnant women interviewed	16
# of currently pregnant women currently attending antenatal care	16/16
# of parous women who had last delivery at home	17/20
# of women who said they prefer to deliver in a facility	9/23
# of women who said they preferred facility delivery who delivered at home at last delivery	6/9
# of parous women who said decision about place of last delivery was their own	6/20
# of parous women who said decision about place of last delivery was ‘God’s’ or chance	11/20
# of parous women who said place of last delivery was the decision of husband or others	2/20
# of parous women who attended a postpartum clinical visit within 1 week after delivery	19/20

Focus groups for TBAs and men were asked similar questions about birth preferences, their roles during pregnancy and delivery, and decision making around place of delivery.

### Decision makers for place of delivery

In response to questions about who decision-makers for place of delivery were, there was not uniform agreement between groups interviewed. One third of female participants expressed strong agency to make their own decision about the place of delivery.

“Safety is the only consideration, I decide. My husband and mother-in-law have no say.”

“ No one decides but me.”

“Me and me alone.” —3 female IDI participants.

Only two female participants expressed a feeling of complete powerlessness to decide:

“I will be controlled by all.” “Even if I say something, I am being told not to do it.” —female IDI participant.

Other women, who did not express a sense of personal agency in the decision to have a home delivery in their last pregnancy attributed the decision to “God”, and explained that the unexpected onset of labor was the deciding factor to give birth at home in their last delivery regardless of personal preference.

“No one decided, I had labor pains then delivered at home. God decides, as facility is far.”

“God plans for me. If it was up to me, I would have gone to hospital.” —2 female IDI participants.

Notably, most women who expressed both preference and agency for a facility delivery, ended up delivering their last child at home because of ‘sudden onset of labor’. While distance and transportation were mentioned by some as reasons they decided not to go, even those who reported living within 30 min walking distance of the clinic decided to have a home delivery once contractions began.

The frequent report of sudden labor led to a follow-up question to women and TBAs about whether they thought a maternity waiting house near a ward clinic would help them get to a clinic on time. Although not all were familiar with the concept, when it was explained, all who were asked affirmed that it would be beneficial to them in their situation. Key informants from ward health facilities, including the ward health officer affirmed this potential solution as well.

Among TBAs there was a general consensus that husbands were the gatekeepers for a facility vs. home delivery. They attributed this to his control of the financial resources needed for transportation, as well as his preference for traditional norms:

“Not this way (facility birth). Maasai men don’t want to incur any cost.”

“A man will deny (facility birth) to his wife because he says others have given birth at home so she can give birth at home too.” —2 TBA’s FGD participants.

From the men’s FGDs, there were a few who affirmed that the husband is the sole decision-maker for place of delivery. Several also expressed an inability to pay the cost of transportation for a facility birth, especially to the tertiary care center in Karatu. More generally, however, there was consensus among men to affirm the role of the TBA in making a judgment about whether a facility birth was necessary based on their expertise about the progress of labor.

“TBAs are the ones to influence where a woman should give birth because they are close to the pregnant mother; I do not even touch her stomach because I am afraid of even being near her. She can tell me that a child is doing well and so she is the one to advise me to take her to hospital or not and I do listen to her because I trust her as I do not decide to take her to the hospital without the TBA’s word.” —Men’s FGD participant.

### Traditional practices in a home delivery

Descriptions of practices around delivery preparation, ANC, and during labor in a home delivery were elicited from pregnant and parous women, TBAs, and husbands, to better understand rituals or services that are desired or expected in a traditional delivery.

#### Antenatal and postpartum clinical care

Pregnant and parous women were asked if they attended ANC visits during their last pregnancy or current pregnancy if they were pregnant. Every woman affirmed that she had attended ANC in the past and was attending ANC in her current pregnancy. TBAs also affirmed that they accompany women to their ANC visits.

Even women who said they preferred to have a home delivery, affirmed the importance of completing ANC visits and believed that they would be told if a hospital delivery was necessary by clinical staff. Several women provided the interviewer with the clinic cards they received showing the number of visits completed and estimated delivery date.

Men also affirmed the value of ANC visits prior to delivery and confirmed that the clinic does not allow a woman to come alone to her first ANC visit, but must be accompanied by her husband so they can provide counselling as well as HIV testing to the couple. This was confirmed by health facility staff except in the case where the woman was unmarried, or the husband was verifiably unavailable. Men’s attitudes also reflected changing norms about the practice of going to antenatal care at a clinic:

“Due to the health education we’re receiving, we normally ask our wives to attend clinic or escort her and do the test together to know if you’re safe. So I advise her to go to the clinic until the time of delivery. In the past, TBAs were our clinics; they were the ones who knew how the child in the belly is doing until delivery.”—men’s FGD participant.

According to nurse-midwives, all three facilities in the ward promote 4 + ANC visits and provide a minimum package of ANC services as defined by MoHCDEC. Midwives also provide counseling on a variety of topics including the importance of a facility delivery.

“We teach them about hygiene after delivery, family planning. We teach them proper breastfeeding. Also we remind them about the importance of facility delivery, and if there is a problem of transportation, we advise a mother to have her own saved bodaboda (motorcycle-taxi) phone number so when she has a problem, instead of waiting for a TBA she can call the bodaboda and come to the facility. We do this when we know the mother is coming for her last ANC visit” —nurse midwife at Nainokanoka clinic.

Women and TBAs were also asked about attitudes toward post-delivery care and if and how long they waited before going to a clinic post-delivery. The majority of women interviewed, who had their previous delivery at home, affirmed that they went to a clinic within 24 h. Only one responded that she had not taken her last child at all to date. TBAs affirmed that it was common practice for them to escort women to a clinic within 24 h of delivery.

#### Home delivery preparations

Notably, every woman, regardless of whether they expressed a preference for a facility or home birth, reported making preparations for a home delivery. Women identified several items for which they were responsible to prepare for a delivery in the home. The most common preparations mentioned were:

- Preparation of butter and ‘jelly’— Preparation of butter to feed the baby was mentioned by almost all women interviewed. Butter is given to the baby right after birth. Key informants explained that it is given just prior to breast feeding because of a belief that it will help the baby digest colostrum better. Women also prepare a local petroleum jelly which is used to rub on the baby’s skin after birth and to massage her abdomen during labor.
- Preparation of firewood— All women prepare firewood for delivery. Key informants explained that firewood was important for cooking during the period after delivery when the mother is nursing and recovering from delivery.

Other items prepared for time of delivery included baby clothes, and several women mentioned food for the time of labor for themselves as well as the TBAs attending them. Foods mentioned included millet porridge, as well as black tea for energy during a long period of labor.

Prior to delivery, a man’s primary responsibility is to find and engage a TBA who will assist with the delivery and accompany his wife during her pregnancy—this includes taking her to ANC visits. TBAs affirmed that they were engaged by men, usually in the first trimester of a woman’s pregnancy. Most men also affirmed that they prepared meat for their wives for the postpartum period—particularly slaughtering a goat. Men also described preparations of meat for the ‘birth ceremony’. Several men mentioned their role in supporting the practice of limiting their wives’ food-intake to assure a low birthweight baby which is considered to be easier to deliver.

“Our preparation starts when she is seven months pregnant, I do tell her to stop doing heavy duties and take some traditional herbs that make her vomit, I do engage a TBA and prepare a goat and a sheep for her.” –men’s FGD participant.

The practice of limiting a woman’s food intake was mentioned by one other husband, specifically describing a role for his mother during pregnancy to limit eating by his wife which could cause a ‘large baby’ that would be difficult to deliver:

“There is one thing that my colleague haven’t said, for us Maasai when you see your wife is pregnant sometimes you may ask your mother to stay with her because it is prohibited for her to eat oily food which may cause a baby to become fat in the mother’s womb. This is because others use operations (episiotomies, c-sections) while we use the natural way. It is important for a woman’s mother or mother-in-law to stay near her so she cannot eat these kinds of food to protect her and the child.” –men’s FGD participant.

Key informants did confirm that the practice of limiting food consumption for pregnant women in the third trimester of pregnancy persists despite recommendations for a balanced diet during pregnancy from nurses during ANC visits.

#### Home delivery procedures

Women giving birth were asked about who was present at a home delivery and every one of them reported being attended by at least one, and up to four TBAs who helped them by preparing food, massaging them, rubbing oil on them, ‘holding them during delivery, ‘pulling out’ the baby, and cutting the umbilical cord. All women reported giving birth in a kneeling or squatting position. Several TBAs reported women giving birth lying on their side, although others described moving to a kneeling position once the woman started pushing. One TBA described in some detail the way that multiple TBAs may work together to support a woman’s birth position when she is pushing during a difficult delivery:

“Giving birth is hard. A mother may be in labor and fail to give birth, so several TBAs might help. The woman kneels on one TBA, and another sits between her legs—she is the main TBA to receive the baby. Others may sit at the back (of the mother).” TBA’s FGD participant.

TBAs reported numerous responsibilities during birth. Those most frequently mentioned were:

- Feeding the mother— This was reported in all focus groups and was considered a major factor in assuring that a mother would have the strength for delivery. Preparation of black tea was also mentioned as a way to give her strength for delivery.
- Massaging the mother
- Receiving the baby, cutting the cord, delivering the placenta
- Cleaning the mother and the baby

All TBAs and women reported that mothers were given the infant immediately for breastfeeding after the child was cleaned, received butter, and rubbed with homemade petroleum jelly. TBAs were aware that breastfeeding helped reduce postpartum bleeding and mentioned it as a reason for giving the mother the baby for breastfeeding immediately. No man reported having a role in the delivery itself, or even being present in the home where delivery was taking place.

Nurse midwives interviewed about facility deliveries were asked about delivery procedures in clinics, particularly their ability to accommodate traditional birth practices. Responses were mixed, with nurse midwives interviewed at the two dispensaries expressing more flexibility around the roles TBAs would play in a facility birth than the midwife at the health center. All clinic midwives reported that TBAs escorting a woman to a clinic for a delivery outside of an emergency, such as obstructed labor or hemorrhaging, was rare. In those cases, patients were immediately transported by ward ambulance to Karatu for comprehensive emergency obstetric care.

In the rare case that a TBA did bring a woman in for a normal delivery, they were not generally invited to participate in the delivery during final stages of labor. At Bulati dispensary, a nurse said they would only allow a TBA to receive the baby after delivery. At Irkeepusi, a midwife said TBAs were allowed in the delivery room and could sometimes help with translating for them from Kiswahili to Maa, as many mothers did not speak Kiswahili. At the Nainokanoka health center, the midwife expressed skepticism about TBAs motives and suspected they were generally trying to ‘learn something they can use for a home birth by watching.’ She also suggested that TBAs represented a barrier to women choosing a facility delivery.

All nurse midwives interviewed also reported that birth position for deliveries in ward clinics was lying down (lithotomy position) and other positions could not be accommodated during the final stage of labor. One midwife did acknowledge one way in which her facility accommodates a traditional preference, which was to respect the Maasai taboo against episiotomies and the use of sutures to repair vaginal tearing during birth.

#### Obstetric emergencies in a home and facility delivery

Several parous women interviewed reported having a difficult delivery, and one was referred to Karatu for a C section in her last birth. Another woman described a bad home delivery experience in which she was held down and her abdomen was pressed down to force the baby out.

“You may give birth and get ill. At home they press your tummy like this. They hold you so you can’t escape, they scare you during delivery.” —female IDI participant.

TBAs were asked about how they contend with complications during a home delivery. Every one of them confirmed that if they were not able to help a mother deliver, they arranged for her to be transported to a ward health facility (from which they could be transported by ambulance to Karatu).

Prior to making this decision, TBAs reported massaging a mother’s abdomen during difficult labor, feeding her porridge and giving her black tea for energy to sustain pushing. One TBA claimed to have experience with successfully delivering babies no matter what the presentation. Another reported at least one unorthodox practice employed during a difficult delivery:

“If the baby has not come out you massage a mother and you put a baby cow beside the mother they may come together. And if you see the baby is coming you remove the baby cow and continue to massage the mother.” -TBA’s FGD participant.

Nurse midwives at clinics reported capacity to deliver basic emergency obstetric services at any hour, 7 days per week. Basic emergency care included repositioning a baby in the case of a breech birth and administering medications for eclampsia as well as oxytocin to prevent hemorrhaging. Other complications are referred to the Karatu hospital by the ward ambulance based at Nainokanoka Health Center.

### Preferences and barriers for home vs. facility delivery

#### Home delivery preference

All female IDI participants were asked where they would choose to have their next child. Focus groups of men and TBAs were also asked the same question. Responses for all these groups were evaluated for convergences and divergences. Just under half of women interviewed expressed a preference for a facility delivery at their next birth. These responses, however, indicated some geographic variances, notably women from Nainokanoka sub-village, (who generally had the highest level of education as indicated by their ability to speak Swahili) preferred facility delivery, while most women in Irkeepusi sub-village said they preferred a home delivery, or did not consider it to be their decision.

Distance was not easy to assess as an access-limiting factor in itself. All women interviewed were asked about the time it would take to walk to a facility for birth; answers ranged from 30 min to 3 h. There was no evident correlation between distance and delivery preference among those interviewed.

Those who reported having ‘no-preference’ when asked about where they would have their next child did not consider themselves to have agency: Either others would make the decision, or the place of delivery would be determined by ‘God’. Leaving the question to ‘God’ was considered a default preference for home delivery (in the absence of a complication during delivery) as it indicated an absence of a birth plan to reach a facility once labor began.

When women, TBA focus groups, and men’s focus groups who preferred home delivery for their next child were asked why, responses generally fell into 3 sub-categories:

1. Preference for traditional rituals and care during a home delivery

Female participants who expressed a preference for home delivery tended to emphasize the kind of care they and the baby received from TBAs. Psychosocial preferences included trust in TBAs and comforts of home, particularly being bathed, massaged, rubbed with petroleum jelly, and fed during the delivery. Lack of familiarity with the facility environment was also an expressed psychosocial barrier, although neither woman who had a facility delivery complained of a negative experience. Others who preferred a home delivery generally had a positive impression of clinics but saw it as a second-tier intervention if there were complications identified during ANC visits or during a home delivery.

Men were also divided in their preference, but those who preferred home delivery, tended to emphasize trust in TBAs, whom they considered to be as competent as a doctor to do a home delivery. Men also trusted TBAs to make a judgment about when transport to a facility would be necessary during a home delivery. One male participant made a specific reference to medical doctors ‘using a sharp object’ (episiotomy) which he felt was harmful to a woman and considered taboo among Maasai in Nainokanoka.

“For me I think it is better for a woman to give birth at home but it is important for her husband to be close to her just in case anything happens. Because if she goes to the hospital the doctor can delay treating her. A doctor can also use a sharp object to take out the baby and it is so painful. But there are women (TBAs) at home who could help her to deliver a child without excessive pain.” –men’s FGD participant.

All TBAs in the focus groups affirmed, in principle, a preference for a facility delivery for which they reported being strong advocates in the face of family gatekeepers (husbands). More probative questions and reports from key informants suggest that TBAs may not be as supportive as they claim, particularly in light of their belief that overcrowding at clinics will result in a woman in labor being turned away.

2) Trust Gap in Facility Capacity

One of the most pervasive reasons given for a preference for home deliveries was overcrowding at facilities in the ward that had reportedly led to women in labor arriving at a clinic and being denied service. TBAs were particularly vocal on this issue and cited the problem of overcrowding in delivery rooms at clinics in the ward overwhelmingly as the reason they resist taking women for a facility birth. According to TBAs, at least 2 women, escorted by TBAs to a clinic, were told there was no space in the delivery room and had to return home only to deliver in the bushes. This story was shared in both TBA focus groups, and in men’s focus groups as well:

“When they have difficulties during birth, we take them to the facility, but a doctor denied one (interrupting voice; Two!) a place of delivery because there was not enough room....We want the facility to be larger with more health workers as now the country is big. We cried on the street that day. The birth attendant helped the mother to give birth in the bushes. It is hard to go to the community as they turn on you because that scandal of a doctor denying a pregnant mother at the facility door has spread. So it is hard to say ‘bring the mother for a facility delivery’ while we don’t have a place of delivery.” –TBAs FGD participant.

While this account was not first-hand and was not reported as a personal experience of any TBA who was present in the focus group, nor any female IDI participant, it is pervasive and does indicate a serious trust gap in facility capacity that warranted follow-up with key informants at all three clinics. Since the ward has averaged less than 5 deliveries per month at each clinic in the past 15 months, the problem of overcrowding in delivery rooms seemed incongruent with the data.

Clinical staff at all three ward facilities were interviewed and asked about the frequency of a woman in labor being turned away because of overcrowding. Midwives at the Nainokanoka health center and Bulati dispensary were not aware of this event ever happening in their facilities during their tenure. The nurse at the smallest dispensary (Bulati) also said that while they did have only one delivery room bed, they could accommodate mothers in labor in other beds in the facility and would not turn anyone away. She speculated that a birth might have happened on the way to a clinic, prior to arrival, but not as a result of being denied a space.

A nurse at Irkeepusi dispensary, however, believed that several women may have been turned away from there at a time in the past when a non-obstetrically trained health worker was covering a shift at the clinic—although not as a result of overcrowding. This was a significant finding. Such a breach of trust for any reason would certainly justify TBA skepticism about taking a woman to a facility for delivery if there was any risk of her being turned away during labor. This experience has likely led to the generalized belief that all clinics in the ward are understaffed and overcrowded.

3) Facilities only for emergencies.

All participants (women, men, TBAs) who preferred a home delivery did consider a facility to be a second tier of assistance if there was difficulty during delivery. Women and men also mentioned relying on ANC visits to identify any potential warning signs and a recommendation for a facility delivery. Men who preferred home delivery acknowledged that going to a hospital was necessary and worth the transport cost in the event of an emergency. They expressed trust in TBAs to make that determination.

“Giving birth at home is my preference. TBAs are the same as doctors, when looking at the delivering woman they will know whether she will deliver safely or not. For a baby that cannot be delivered safely, TBAs would notice as early as possible and if there is a need of taking her to the hospital they recommend and advise us what is to be done. Therefore, we would like these TBAs to continue as caregivers for pregnant women while at home as long as they do go to the (antenatal) clinic until the day of giving birth.” –men’s FGD participant.

Health facility key informants noted that the practice of TBAs bringing women to the facility when they failed to deliver was the most common reason they came to a facility, and by that point, they were usually referred and transported to Karatu for comprehensive care.

Accessing facilities in the event of an emergency is consistent with findings that men, women, and TBAs do access the health care system and depend on ANC visits to give them information on the progress of the pregnancy and advise on risks. Other factors mentioned by men, to a lesser extent, were cost of transportation, although it was not always clear whether they were talking about going to a ward clinic or to the tertiary hospital in Karatu when they referred to it during focus group interviews.

### Factors that have led some women (or key family decision makers) to change their delivery preference to a facility delivery

#### Facility delivery preference

Responses about preference for a future delivery indicate that there is a slow trend toward adopting facility delivery in principle. Specific reasons most-often cited by those who expressed a preference for a facility delivery were grouped into several sub-categories:

1. Changing Norms

Women, men and TBAs all acknowledged awareness of changing norms and a push by local health facilities and the EbOO project to encourage facility birth, especially during ANC visits. Several men referred to the way things have changed since the time of their fathers. TBAs and men’s focus groups were aware that there are expectations that women should deliver in clinics to be safe and some men acknowledged this change need not impede or contradict traditional practices surrounding a birth.

“Traditions and customs won’t prevent a woman from going to hospitals to give birth and even the traditional ceremonies can be done as soon as she comes back from the hospital usually after seven days.” —men’s FGD participant.

2) Fear of ‘new’ complications

Several women, TBAs, and men, mentioned the need for facility delivery in light of ‘new’ complications that women experience nowadays. New diseases were mentioned as well, particularly HIV. Perceptions of new complications is likely a result of an increased awareness of risk and susceptibility rather than an increase in new risk factors in the recent past.

“A hospital is a good and safe place. In the past we were ignorant. We did not have anything to say. Now there are so many diseases and complications.” – men’s FGD participant.

“The world has changed not like past days. There are many risks of diseases and complications during and after delivery so it is better for me if I go to the hospital” – female IDI participant.

3) Pain relief and control of bleeding

Several women as well as men specifically mentioned the benefit of receiving an injection for pain relief, as well as the ability to control bleeding and fully remove the placenta at the hospital as a reason why facility delivery is preferable.

“In hospital ... after delivery they clean you and they inject you with a syringe so you can not feel the pain and you are cleaned quickly. You can even wear your underwear right after birth. In a village it is not possible to clean you completely, you may have a discharge for over a month.” — female IDI participant.

4) Bad experience in a home delivery

A few women expressed a preference for facility deliveries in light of bad experiences in a previous home delivery. Excessive bleeding was the number one concern.

“You may give birth and get ill…or the placenta won’t come out and they cannot do anything (at home) only to breastfeed the baby. Because if the placenta delays coming out, it is a disaster for mothers. That is why we like to give birth at the hospital” –female IDI participant.

## Discussion

This study was undertaken to better understand the reasons for the rate gap between Maasai women who complete ANC and those who return to a facility for delivery. Specifically, research questions focused on understanding and documenting: 1) who decides place of delivery, 2) what non-medical practices are part of a traditional home delivery, 3) what are barriers and preferences regarding facility vs. home delivery, and 4) what factors have led some women (or key family decision makers) to change their delivery preference from a home to a facility delivery.

Findings are consistent with Magoma [8], and Rogeveen [9] nearly a decade ago, who described high ANC attendance and low utilization of skilled care in Ngorongoro crater region as a ‘complex reality’, based on access barriers as well as preferences for traditional practices. In this study we found that psychosocial preferences as well as access barriers continue to play a significant role in explaining the lack of facility births in Nainokanoka ward. Distance, as well as cost and availability of transportation were themes elucidated by all groups. Preference for ‘comfort’ services offered by trusted TBAs such as massaging the abdomen with oil and feeding during delivery were also highlighted by those who preferred to have a birth at home even after receiving counselling on advantages of a facility birth during ANC. These findings are consistent as well with findings from similar groups in Southern Tanzania [23], Kenya [11], and Zambia [15]. Rapid onset of labor was also mentioned frequently as a factor for delivering at home, a finding also supported by Kubani et al. in Malawi [13].

Despite these preferences and barriers, the EbOO project has seen an increase in adopters of facility births in the past 15 months since the project began and this study has documented factors that have reinforced as well as impeded this progress.

### Decision makers for place of delivery

Most women interviewed reported having some agency to decide the place of delivery; and one third of those who were currently pregnant expressed a preference for a facility delivery for their next birth. This finding was surprising given the rigid patriarchal structure of Maasai society in Ngorongoro district [27]. Despite this reported preference, it was observed that all but one of the participants had a home birth at their last birth because the decision was ultimately ‘made by God,’ in that labor came on unexpectedly and they did not believe they could make it to a clinic on time.

TBAs attribute decision-making power to men primarily because they hold the financial resources necessary for transport to a clinic. Men’s focus groups acknowledged that one decisive factor was the cost associated with birth, for which they were responsible. However, on the question of decision-making for place of delivery, responses were mixed. Many, but not all, expressed a preference for home delivery, but also affirmed that the final decision about place of delivery fell with the TBA. They trusted the TBA to advise them on whether a facility birth was necessary because of a complication identified during ANC visits, or during the delivery itself. Based on all interviews, it is evident that TBAs do have significant influence on the decision about where to give birth. They are trusted by husbands and spend 3 to 7 months with women during their pregnancy. While TBA focus group participants affirmed their commitment to promoting facility births, practice suggests otherwise, with nurse-midwives at clinics reporting that it was rare to see a TBA accompanying a woman to a facility for a normal delivery.

### Traditional practices in a home delivery

All pregnant women interviewed reported making preparations for their next birth consistent with a home delivery. Women prepare a home-made petroleum jelly to rub on the baby which is also used by TBAs to massage laboring women during delivery. They also prepare butter to give the baby, and store firewood for the post delivery period during which they rest and breastfeed. Based on comments from the men’s focus group and confirmed by key informants, the practice of limiting a woman’s food intake and giving herbs to induce vomiting during the last trimester to insure a ‘small baby’ persists, and has been documented by others as well [9]. Several men identified their mothers as instrumental in enforcing this practice.

Only women are present at the delivery, including 1 to 4 TBAs who help with the birth by supporting the woman in her birthing position and receiving the baby. They also cut the umbilical cord, remove the placenta, wash and rub jelly on the baby, then return it to the mother for breastfeeding. All TBAs and women reported that babies were delivered in a kneeling or squatting position. If women are having difficulty during labor, TBAs will massage them with oil, and feed them or give them black tea to drink for energy.

Women also reported giving their baby butter prior to first feeding. Key informants also reported that this feeding usually does not happen more than one time.

Clinic midwives confirmed that birth positions other than lying on the back could not be accommodated in a facility birth. They also discourage the practice of giving butter prior to breast feeding and in two of the three clinics, nurses said that they did not give a TBA a role in delivery during labor but allowed them to receive the baby once born. Nurse midwives did say they made some accommodations for traditional taboos by not performing episiotomies or suturing small vaginal tears after delivery.

Several practices mentioned in preparation for and during traditional birth do raise serious health concerns, particularly the practice of reducing food for pregnant mothers in the third trimester, which presents risks both for mother and the child. Feeding an infant butter also poses risks as well and is not consistent with World Health Organization (WHO) protocol on exclusive breastfeeding for the first 6 months.

### Preferences and barriers for home vs. facility delivery

Women who preferred a home delivery mentioned familiarity and the comforts of home and traditional care modalities as reasons. Nurse midwives acknowledged that clinics were not willing or able to accommodate certain traditional birth practices, including allowing the TBA to actively participate in the delivery, yet nearly half of the women interviewed said they would prefer a facility delivery for their next birth. Two women interviewed who had had a facility delivery reported having a very positive, even life-saving experience.

The chief barrier to choosing a facility delivery for women who said they would have preferred one during their last delivery was the ‘sudden onset of labor,’ which prevented them from going to a clinic. Attributing this entirely to distance from facility or lack of transport fees is difficult, because several women who reported this were less than an hour from a clinic on foot. It is possible that this explanation was a simplification of the underlying reality of strongly-held norms that women were not ready to fully disclose during an interview.

One care-group-based solution to this issue for women who want a facility birth is more intentional promotion of a birth plan, a recommendation supported by Magoma et al. [8]. The suggestion of constructing a maternity waiting house in the ward, an intervention supported by the WHO which has shown success in similar settings [10], was also received favorably by those asked because it would allow them to be near a clinic prior to onset of labor.

TBAs have significant influence over their female clients as well as their husbands in deciding place of delivery. They affirmed their support of facility delivery in principle but expressed reticence to take women to clinics because of ‘overcrowding’ and the risk of mothers-in-labor being turned away and forced to give birth in the bushes. Accounts of two women in labor being turned away at the door of a clinic are pervasive in the community and are given as a prime reason for lack of trust. A midwife confirmed that this did happen at Irkeepusi dispensary due to lack of properly trained staff in the past, but that the problem has been mitigated. The lack of trust that resulted, however, appears to be a significant deterrent to adoption of facility delivery in the entire ward, and has resulted in a generalized perception that all clinic delivery facilities in the ward are overcrowded (despite data to the contrary). Perhaps direct meetings between EbOO project TBAs and health facility staff to rebuild trust and mutual respect facilitated by the NDI would be a place to start (presuming that the staffing issues that resulted in this problem have been fully resolved).

In addition to direct meetings, sensitivity trainings could be offered to facility nurses on cultural norms in the ward and the significant role TBAs play in traditional deliveries. A strategy for developing a uniform protocol for TBAs roles in a facility delivery, understood by all facility staff, could help to resolve the issue of facility nurse distrust of TBAs and is supported by several systemic reviews on TBA-facility partnerships [28, 29].

Cost was mentioned by men as a barrier to a facility delivery, and although delivery is free at clinics, transportation is a cost. The cost of transport cited by men during the FGDs was for a dala-dala (public taxi) ride to the hospital in Karatu, which would be significantly more than a boda-boda (taxi-motorcycle) ride to a local clinic. The study design did not include an assessment of income and the extent to which it was correlated to preference for facility delivery. The availability of boda-bodas in the ward was also not assessed in this study. In light of the fact that all women interviewed were regularly accessing clinics for ANC, it is hard to attribute transport costs as the primary barrier to women giving birth at a facility in the ward. Although without a birth plan, it could be a significant factor.

Based on attitudes and practice of study participants, it appears that facilities are seen by most as a second-tier intervention for delivery and accessed only in the event of complications during a home delivery. This mindset raises red flags, as delay in deciding to access care can contribute significantly to maternal and newborn morbidity and mortality.

### Factors that have led some women (or key family decision makers) to change their delivery preference to a facility delivery

Despite a trust gap that has developed among TBAs about facility capacity, and the perception by many that a health facility is a place of last resort in the event of an emergency, there is also clear evidence of a change in norms. Based on clinical data collected from clinics for the EbOO project, facility births in the ward have increased from 2% at project baseline, to nearly 30% over a 15-month period. Attribution of this behavior change is challenging because there are multiple actors advocating for facility delivery at the ward level and there is no comparison group in a comparable setting where care groups are not being implemented.

It is evident from men’s focus groups and interviews with individual women that there is awareness of the availability of facilities for delivery and the advantages they offer. This information is provided in care groups but also comes from health workers during ANC visits who have confirmed that all women receive counselling on the advantages of a facility delivery. Men, who are required to go to their wives’ first ANC visit during a pregnancy receive this information as well. The perception of increased risk of ‘new complications’ is likely an expression of increased awareness of susceptibility to poor home delivery outcomes. All these factors are contributing to changing norms.

The EbOO project has enlisted TBAs as care group volunteers and have provided training and incentives for them to promote facility births and 4 + ANC visits to women who they visit in their neighborhood groups. Given the significant influence TBAs have with husbands, as well as their pregnant clients, it is reasonable to attribute some of this success to the project theory of change, especially given the rapid rate increase in the 15 months since project initiation. While the gap between 4+ ANC visits and facility deliveries has remained throughout the life of the project, both have increased proportionally and are converging slightly (1.7% increase per month for 4 + ANC visits and 2.1% increase per month for facility births).

### Study limitations and caveats

Any study crossing cultural and language barriers faces some risk of misinterpreting responses. Care has been taken to ensure accurate translation of Maa interviews into English with two translators but errors in interpretation are possible. Key informants have reviewed responses to evaluate their consistency with their knowledge and experience, and effort was made to minimize this kind of error. It is also not always easy to tell if responses reflect the true motivations or feelings of an individual, or if they are trying to ‘please’ the interviewer. Care was also taken to have Maa speaking interviewers who are known to the community, but to some extent the formal questioning, probing, and recording can have unknown effects on a participant.

The scope of the study also did not include extensive analysis of known confounders such as education and income level in evaluating the responses. The focus of the study was to understand what decision-makers’ attitudes and preferences were factors in choosing a place of delivery, and why home delivery remained a strong preference for women in this community who complete ANC visits.

The timing of interviews was relatively short and provided a cross-sectional rather than longitudinal analysis of changes in norms. There was no baseline data for an individual woman’s preference and no way to evaluate the changes she made about a delivery decision over the course of her pregnancy.

Finally, the EbOO project is a behavior change based intervention and most solutions are focused on changing behavior of participants rather than finding systemic solutions at the health systems level. This is due to the practicality of what a community-based intervention can do to promote best practices in the context in which it is working.

## Conclusion

This qualitative study was conducted in Nainokanoka ward of Tanzania to better understand the rate gap between Maasai women who complete ANC visits and those who return to a facility for delivery. Results show that there are several factors that may account for this anomaly. 1) While many women express agency to decide on place of birth, more than half prefer traditional services provided by a TBA in her own *boma* (home). Most who expressed a preference for facility delivery still gave birth at home citing ‘sudden onset of labor’ as the deciding factor, indicating a gap between intention and behavior. 2) TBAs, who are potential advocates for facility delivery and have significant influence over decision-making gate keepers (husbands), identified a trust gap in facility capacity based on the bad experience of two women who were turned away at the time of labor. 3) Community members generally see a facility delivery as a second-tier intervention to be used only when a complication is identified during ANC or an obstetric emergency arises in a home delivery. Addressing these three issues are key to increasing the use of facilities during delivery.

One intervention discussed with community members during interviews to mitigate the problem of distance and traveling during labor, was the construction of a maternity waiting house at the centrally located ward health center. Reception to this idea was positive among stakeholders and could directly address the reported problem of women who would prefer a facility delivery but are not able to get to one once labor has begun.

Addressing the trust gap between TBAs, who are potential advocates and community influencers, and ward health facility professionals regarding capacity and staffing at ward facilities could be facilitated by NDI, the EbOO project implementing partner. If all clinics are now fully staffed to handle deliveries at any time, that needs to be communicated effectively to those who have significant influence in decision-making about place of delivery, particularly TBAs. Further sensitization of facility nurses on the psycho-social support role TBAs play in the lives of pregnant women in the ward could also be beneficial in developing mutual respect and trust between TBAs and nurses. Collaboratively developing clearly defined roles for TBAs in a facility delivery implemented across all ward facilities could also address facility nurse mistrust of TBAs and encourage TBAs to support facility births more fully.

Clinical data collected since the start-up of the EbOO project does indicate that care groups are having an attributable impact on the increasing the number of women completing ANC visits and having facility deliveries in Nainokanoka ward. Increased understanding of factors affecting decision-making will hopefully lead to further success in closing the gap between women accessing ANC services and those accessing facilities for delivery.

## Data Availability

The datasets used and/or analyzed during the current study are available from the corresponding author on reasonable request.

## Abbreviations

ANC: Antenatal Care
ART: Antiretroviral Therapy
DHS: Demographic and Health Survey
EbOO: Ebiotishu Oondomonok Ongera (“Healthy Mother and Child” in Maa language)
FG: Focus Group
FGD: Focus Group Discussion
HPC: Household and Population Survey
IDI: In Depth Interview
MCC: Mennonite Central Committee
MDG: Millennium Development Goals
MMR: Maternal Mortality Ratio
MoHCDEC: Ministry of Health, Community Development, Gender, Elderly and Children
MOHSW: Ministry of Health and Social Welfare (now MoHCDEC)
NCA: Ngorongoro Conservation Area
NDI: Naiboisho Development Initiative
RMNCH: Reproductive, Maternal, Neonatal, and Child Health
TBA: Traditional Birth Attendant
WHO: World Health Organization

## Footnotes

1 Women’s actual age range is not known as15 of 23 women interviewed did not know their precise age.
